# Supplementary material for: Content-rich biological network constructed by mining PubMed abstracts
Source: BMC Bioinformatics. 2004 Oct 8;5:147. doi: 10.1186/1471-2105-5-147 (PMC528731; doi:10.1186/1471-2105-5-147)
Supplement: Additional File 2 — The original results of the above study (non-essential files are deleted to keep the file size under the limit set by BMC bioinformatics). [file 1471-2105-5-147-S2.bz2 › chilibotAdditionalFile2/dip05/134ID7935795E479/html/YWHAB_RAF1.html]

 


 **YWHAB** and **RAF1** 
  
Found 8 abstracts in PubMed, retrieved 05.  
 

 What does Google say? 
 PDF only 
| .edu only 

---

**Interactive relationship** (e.g. stimulation, inhibition, etc)

**Inhibitory relationship**- The colony forming ability of NIH 3T3 cells overexpressing 14 3 3 beta  [ **YWHAB** ]  in soft agar medium was efficiently abolished by exogenous expression of a dominant negative mutant of MEK1 and 14 3 3 beta  [ **YWHAB** ]  physically interacted with Raf 1  [ **RAF1** ]  in these cells.  Ref: 11062170 Carcinogenesis, 2000
**Stimulatory relationship**- These findings indicate that 14 3 3 beta  [ **YWHAB** ]  has oncogenic potential, mainly through enhancement of Raf 1  [ **RAF1** ]  activation and resultant augmentation of signaling in the MAPK cascade.  Ref: 11062170 Carcinogenesis, 2000
**Neutral relationship**- Second, Bcr does not bind to Raf  [ **RAF1** ]  directly in the two hybrid system, but co expression of 14 3 3 beta  [ **YWHAB** ]  allows complex formation.  Ref: 7588613 EMBO J, 1995
